# Dermatitis Venenata

**Published:** 1907-12-28

**Authors:** Louis E. Stamm


### THE GENERAL PRACTITIONER'S COLUMN.

[Contributions to this Column are invited, and if accepted will be paid for.]

### DERMATITIS VENENATA.

By Dr. LOUIS E. STAMM, M.D., B.A., B.Sc.

Dermatitis Venenata may present itself in a variety of forms, and its causes are so multifarious that they often require the most careful inquiry and search in order to discover them. It has to be distinguished chiefly from various forms of eczema and from urticarial rashes, with any of which it may frequently be confounded. Now, the essential condition which differentiates such a dermatitis from other skin lesions is that the exciting cause is external to the body, and affects certain definite areas of skin exposed to its influence. It is most important, therefore, to observe the area of distribution of the lesion. It not infrequently happens that chemicals used in the dyeing or dressing of materials may set up a dermatitis in susceptible individuals wherever the materials come into immediate contact with the skin. A case may be cited in this connection in which an irritating erythematous dermatitis over the upper part of the chest and arms was traced to a bodice which had been recently dyed black. The area of skin affected exactly corresponded to the parts where the underwear failed and the dress came into immediate contact with the skin.

A dermatitis which is confined to the hands is the most usual form met with, and it generally requires but a little careful inquiry into the occupation of the patient to elucidate the cause. A peculiar feature of these morbid conditions that must always be borne in mind is the idiosyncrasy of the individual. With those who have an inherent predisposition to eczema exposure to any sort of irritating chemical may act as an exciting cause and effect a recrudescence of the trouble, but apart from this it is not uncommon to find individuals exhibiting a peculiar intolerance to certain chemicals which are not ordinarily credited with specially irritating properties.

There is a variety of dermatitis venenata which is likely to give the clinician more trouble in diagnosis than any other. It is that which results from contact with some poisonous or irritating plant. The list of plants which may in individual cases set up such a condition is almost inexhaustible. A comprehensive account of these plants is given in a monograph on the subject by Dr. J. C. White, of Boston. In America the Rhus venenata and toxicodendron, or poison ivy and oak, are the most common delinquents, whereas in this country the *primula obconica* has the first place of honour in this respect. The poison is supposed to consist of a volatile acid, which resides in the hairs of the plant.

Soon after exposure an intense itching and sense of heat is felt in the part affected, and in two to twenty-four hours afterwards the eruption appears, which may be papular or erythematous. Occasionally vesicles or bullæ may be formed, and the eruption may even become pustular. There may be constitutional disturbance in severe cases with a rise of temperature. The condition generally subsides in the course of a few days if there is no further exposure to the plant, but it may last two or three weeks. Generally only the hands are affected from handling the plants, but in highly susceptible individuals the face may be involved, probably by autoinoculation from the hands, and in a similar manner other parts of the body also may show the lesion, a fact which is specially liable to lead to confusion and error in diagnosis.

A recent case of dermatitis from poisoning with the *primula obconica* is worth placing on record, both on account of the severity of the condition and also on account of the length of time during which the patient suffered before the nature of the affection was detected. The patient was a lady whom I was first called in to attend in the early spring of the present year. She was then suffering from an erythematous rash over the face and hands. There was considerable swelling of the parts affected. The eyes were suffused and the eyelids œdematous. There were in addition small vesicles scattered about over the erythematous patches. The patient complained of an acute burning pain and great irritation. She gave the following history of the condition: She had had repeated attacks of a similar nature for the past year, but had been much freer from them during the recent winter months than through the previous spring and summer, when she suffered from the eruption almost continuously. When the trouble first appeared a year ago she consulted a doctor in her neighbourhood, and she was treated for erysipelas. The attack subsided only to recur, and she then sought advice at one of the London hospitals, and remained under treatment for several months. The condition was apparently regarded as eczema, and the patient was placed on a rigid diet and treated with lotions and physic.

I accepted this diagnosis provisionally, and treated her in a similar manner, and as her temperature was raised somewhat I kept her in bed. The attack subsided in the course of a few days. As soon, however, as she was allowed to get about and resume her household duties she was again a victim to the trouble. This occurred two or three times, and it then became obvious that the attacks were due to some cause which did not operate as long as she was kept quiet and confined to her room. The fact that only the exposed parts of the body were affected suggested further some external irritant as the cause. I asked to be allowed to inspect the premises, and immediately discovered at the back of the house a small greenhouse full of the *primula obconica*! I then learned that the patient had first commenced to cultivate these plants in the previous spring, when she had her first attack, and as soon as she was allowed out of her room her first care and attention were given to her cherished plants. Needless to say, the plants were promptly confiscated, and the malady disappeared, and has not been heard of since

